# The relationship between gadolinium enhancement and [18 F]fluorothymidine uptake in brain lesions with the use of hybrid PET/MRI

**DOI:** 10.1186/s40644-024-00761-0

**Published:** 2024-08-19

**Authors:** Tomáš Rohan, Petr Hložanka, Marek Dostál, Tereza Kopřivová, Tomáš Macek, Václav Vybíhal, Hiroko Jeannette Martin, Andrea Šprláková-Puková, Miloš Keřkovský

**Affiliations:** 1 Department of Radiology and Nuclear Medicine, University Hospital Brno, Jihlavská 20, Brno, 625 00 Czech Republic; 2 Department of Radiology and Nuclear Medicine, Medical Faculty, Masaryk University, Brno, 625 00 Czechia; 3 Department of Biophysics, Medical Faculty, Masaryk University, Brno, 625 00 Czechia; 4 Clinic of Neurosurgery, University Hospital Brno, Brno, 625 00 Czechia; 5 Clinic of Neurosurgery, Medical Faculty, Masaryk University, Brno, 625 00 Czechia

**Keywords:** PET/MRI, [^18^F]FLT, Brain tumour, Tumour-like lesions, High grade, Low grade

## Abstract

**Background:**

To evaluate and compare the diagnostic power of [^18^F]FLT-PET with ceMRI in patients with brain tumours or other focal lesions.

**Methods:**

121 patients with suspected brain tumour or those after brain tumour surgery were enroled in this retrospective study (61 females, 60 males, mean age 37.3 years, range 1–80 years). All patients underwent [^18^F]FLT**-**PET/MRI with gadolinium contrast agent application. In 118 of these patients, a final diagnosis was made, verified by histopathology or by follow-up. Agreement between ceMRI and [^18^F]FLT-PET of the whole study group was established. Further, sensitivity and specificity of ceMRI and [^18^F]FLT-PET were calculated for differentiation of high-grade vs. low-grade tumours, high**-**grade vs. low**-**grade tumours together with non-tumour lesions and for differentiation of high-grade tumours from all other verified lesions.

**Results:**

[^18^F]FLT-PET and ceMRI findings were concordant in 119 cases (98%). On closer analysis of a subset of 64 patients with verified gliomas, the sensitivity and specificity of both PET and ceMRI were identical (90% and 84%, respectively) for differentiating low-grade from high-grade tumours, if the contrast enhancement and [^18^F]FLT uptake were considered as hallmarks of high-grade tumour. For differentiation of high-grade tumours from low-grade tumours and lesions of nontumorous aetiology (e.g., inflammatory lesions or post-therapeutic changes) in a subgroup of 93 patients by visual evaluation, the sensitivity of both PET and ceMRI was 90%, whereas the specificity of PET was slightly higher (61%) compared to ceMRI (57%). By receiver operating characteristic analysis, the sensitivity and specificity were 82% and 74%, respectively, when the threshold of SUVmax in the tumour was set to 0.9 g/ml.

**Conclusion:**

We demonstrated a generally very high correlation of [^18^F]FLT accumulation with contrast enhancement visible on ceMRI and a comparable diagnostic yield in both modalities for differentiating high-grade tumours from low-grade tumours and lesions of other aetiology.

**Supplementary Information:**

The online version contains supplementary material available at 10.1186/s40644-024-00761-0.

## Background

Diagnosis of brain tumours is usually based on brain MRI with intravenous administration of contrast agent [1]. Although current guidelines do not provide specific recommendations on the routine use of PET in brain tumours, PET is commonly used as an adjunct to provide insight on tumour characteristics and dynamics for effective patient management. PET is particularly valuable in the assessment of tumour burden and anticancer treatment response, for example, by facilitating identification of predictive factors that may impact patient outcomes or by differentiating treatment-related changes from disease progression [2].

The use of radiolabelled glucose analogue [^18^F]fluorodeoxyglucose (FDG) is limited due to its uptake in normal brain tissue, which can potentially hamper the imaging of gliomas [3, 4]. A better contrast between tumour and brain tissue is provided by radiolabelled amino acids, which are predominantly taken up by tumour tissue [1]. The most commonly used radiolabelled amino acids are [*S*-methyl-^11^C]-*L*-methionine (MET), 3,4-dihydroxy-6-[^18^F]-fluoro-*L*-phenylalanine (FDOPA), and *O*-(2-[^18^F]-fluoroethyl)-*L*-tyrosine (FET) [1].

Another option for brain tumour imaging is the use of a radiolabelled analogue of the nucleoside thymidine [5–7]. Radiolabelled fluorothymidine ([^18^F]fluorothymidine, [^18^F]FLT) has originally been proposed as a marker of cell proliferation that is trapped in cells after phosphorylation by thymidine kinase in the *S*-phase of the cell cycle [8]. [^18^F]FLT does not accumulate in normal brain tissue and thus can be useful in detecting high-grade gliomas (HGG) [3] and brain metastases [9] or in differentiating between high-grade and low-grade gliomas (LGG) [5, 10]. However, the increasing number of recent studies suggest that [^18^F]FLT uptake in brain tissue may be more related to transport through a disrupted blood-brain barrier, [11, 12] similar to intravenous contrast agents on MRI, rather than to the proliferation rate itself. Therefore, there may be close correlation between areas of [^18^F]FLT uptake and gadolinium enhancement, bringing the additional value of [^18^F]FLT-PET into question.

There are a number of studies evaluating the significance of brain [^18^F]FLT on PET/CT for both primary tumours and metastases [9, 13, 14]. Furthermore, the significance of [^18^F]FLT and contrast-enhanced brain MRI performed separately has been compared in a relatively small group of patients [15]. However, as far as we were able to establish, no study to date has analysed [^18^F]FLT-PET and MRI performed simultaneously within PET/MRI examination of the brain.

In this study, we aim to analyse PET/MRI data to assess the relationship between contrast-enhanced MRI (ceMRI) and [^18^F]FLT uptake, and to establish the diagnostic power of both modalities in differentiating high-grade tumours from low-grade tumours and lesions of different aetiology.

## Methods

This retrospective study included patients who underwent PET/MRI examinations of the brain for suspected or known brain tumour, including patients after brain tumour therapy, who met the common indication criteria for PET/MRI examination in a university hospital between July 2017 and December 2022. This study was conducted in accordance with the principles of the Declaration of Helsinki and was approved by the Institutional Review Board (04-160222/EK; 16 February 2022). Examinations with significant image artefacts or missing data were excluded.

### Protocol of [^18^F]FLT-PET/MRI examination

The examinations were performed on a 3.0 T PET/MRI system (Signa, GE Healthcare, Illinois, USA). The MRI examination covered the whole brain using a 19-channel head and neck coil. Pre- and post-contrast axial 3D T1-weighted gradient-echo imaging at isotropic resolution were evaluated to identify contrast enhancement. The amount of intravenously administered gadolinium contrast agent (Dotarem, Guerbet, France) with a concentration of 0.5 mmol/ml was based on the patient’s weight (0.1 mmol/kg), ranging from 2 to 20 ml. The comprehensive protocol can be found in supplement material (Supplement A Table 1).

The PET examination was performed in a single step with an axial FOV of 25 cm and an acquisition time of 40 min. The radiopharmaceutical used was [^18^F]fluorothymidine at an activity of 3 MBq/kg (range 20–340 MBq). PET imaging began as soon as possible after administration of [^18^F]FLT. Images were reconstructed using the 3D OSEM (ordered subset expectation maximisation) method with TOF (time of flight) and PSF (point spread function, VPFX-S), employing 28 subsets and 2 iterations. The reconstructed PET image had a FOV of 30*30 cm with a matrix of 256*256. Atlas-based attenuation correction was applied with 3D GRE Dixon images to create a pseudo-CT image.

A detailed description of the PET/MRI acquisition parameters is provided in the Supplement A Table 1.

**Fig. 1:**
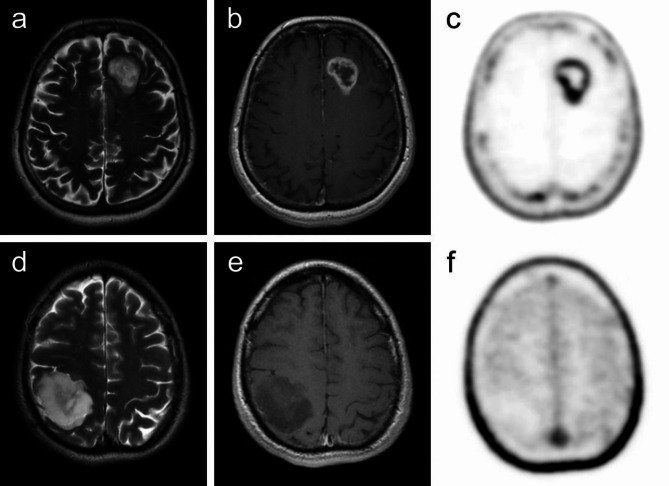
True positive PET/MRI with [^18^F]FLT in a patient with glioblastoma grade IV, IDH wildtype (**a**-**c**) and a true negative finding in a patient with low-grade glioma (diffuse astrocytoma grade II, IDH mutant) (**d**-**f**). A high grade glioma located in the left frontal lobe is heterogeneously hyperintense on T2-weighted image (**a**), similar to a low-grade glioma in the right parietal lobe (**d**). As expected, the high-grade tumour demonstrates strong, mostly peripheral enhancement on contrast-enhanced T1-weighted image (**b**) and high uptake of [^18^F]FLT on PET (**c**); conversely, no enhancement (**e**) or [^18^F]FLT uptake (**f**) can be seen in a patient with a low-grade glioma

### Evaluation of [^18^F]FLT-PET/MRI

All examinations were evaluated by two experienced nuclear medicine physicians and two radiologists, who visually analysed the images and consensually recorded the presence of pathological [^18^F]FLT uptake and contrast enhancement visible on ceMRI.

For the comparison of ceMRI and increased activity on PET, only the localisation of contrast enhancement and increased metabolic activity were considered; neither the extent nor the character of contrast enhancement was taken into account.

For the purpose of the study, the maximum standardised uptake value (SUVmax) was assessed according to EANM procedure guidelines by drawing a region of interest (ROI) around each lesion [16]. For multiple lesions, only the highest value of SUVmax was used for further analysis. The spatial correspondence of PET and MRI was visually verified and corrected by registration if necessary to ensure correct ROI placement. The ROI was drawn over the tumour or tumour-like lesion on the MR image and copied to the PET image. The mean SUV for normal brain tissue was assessed by mirroring the ROI from the suspected lesion to the contralateral brain tissue while maintaining its size. All ROIs were drawn by the same board-certified radiologist and were confirmed by a board-certified nuclear medicine physician. Tumour-to-normal (T/N) ratios were determined by dividing the SUVmax of the tumour by the SUVmean of the normal brain tissue [15]. Image analysis was performed in AW server 3.2 Ext. 4.6 (GE Healthcare, Illinois, USA).

### Outcome measure

To analyse the concordance rate of contrast enhancement on MRI and increased [^18^F]FLT-PET activity in suspected or known brain tumours regardless of their aetiology. Furthermore, the sensitivity and specificity of PET activity and contrast enhancement present on ceMRI in differentiating HGG from LGG and lesions of other aetiology were calculated in a subgroup of verified lesions.

The gold standard for determining the sensitivity and specificity was histopathological examination of resection/biopsy or follow-up by MRI for at least 1 year in case of stable disease, thereby differentiating tumorous from nontumorous aetiology (e.g. inflammation, pseudoprogression or postoperative changes).

The diagnosis of low grade or high grade tumour (Fig. 1) was made either by histopathological confirmation after PET/MRI (*n* = 30) or on the basis of histopathological analysis performed before PET/MRI (*n* = 34), taking into consideration the reported completeness of resection, the character of residual tissue in relation to preoperative findings, information about subsequent oncological treatment, and also the temporal evolution of the lesion to rule-out the treatment-related changes.

**Fig. 2:**
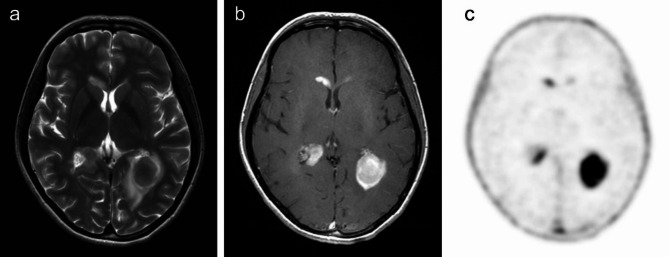
[^18^F]FLT-PET/MRI in a patient with an atypical variant of a tumefactive multiple sclerosis verified by a biopsy. Multiple contrast-enhancing lesions (**b**) with relatively low signal intensity on a T2-weighted image in the axial plane (**a**) are present in the periventricular white matter of both hemispheres, the largest one on the left side near the trigone of the lateral ventricle with a hyperintense rim of perifocal oedema. All lesions demonstrate very high uptake of [^18^F]FLT (**c**). PET examination in this case failed to rule out tumorous aetiology (e.g. lymphoma), which was considered due to a very atypical MRI finding

Pseudoprogression was defined as a new or enlarging area of contrast enhancement occurring early after the end of radiotherapy, in the absence of true tumour growth, which subsides or stabilises without a change in therapy [17]. Postoperative changes were considered as contrast-enhanced streaky areas that remained stable during follow up. A lesion that progressed in size despite maximal conservative treatment was considered as a tumour. Tumours without histopathological verification or without a specified grade in the histopathological findings were reported as tumours of unknown grade. Inflammation was confirmed either histopathologically or in patients without a known tumour with a brain lesion on MRI that did not progress during follow-up or resolved spontaneously with non-oncological treatment (Fig. 2).

### Statistical methods

Statistical analysis included descriptive statistics of basic patient characteristics (age, sex, clinical data). The association between ceMRI and [^18^F]FLT-PET was tested using Cohen’s weighted kappa for all patients, regardless of pathology. To evaluate the effect of pathology on the agreement between ceMRI and [^18^F]FLT-PET, the Fisher exact test with post-hoc analyses was used. Differentiation of HGG and LGG, HGG and non-tumour lesions, and differentiation of HGG from other verified lesions (excluding tumours of unknown grade) were evaluated using Student’s t-test. Sensitivity and specificity were calculated using 2*2 tables. Receiver Operating Characteristic (ROC, Youden’s method) analysis was performed to set the threshold of SUVmax and T/N ratio for differentiation between HGG and LGG, HGG and non-tumour lesions, as well as HGG and all other lesions combined. All statistical tests were performed at a significance level of alpha = 0.05 and by R (R Core Team, 2020) and RStudio (Rstudio Team, 2020).

## Results

**Fig. 3:**
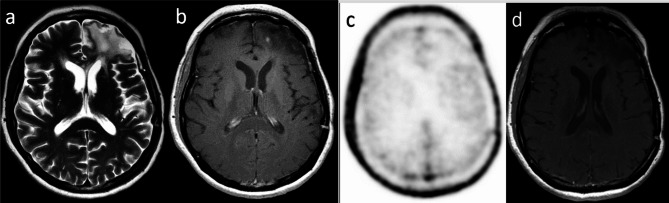
[^18^F]FLT-PET/MRI in a patient with low grade glioma after complex treatment (partial resection, chemotherapy and radiotherapy). In the area of post-treatment changes (**a**) in the left frontal lobe, two years after the last treatment, there was a new spot of contrast enhancement (**b**) without increased PET activity (**c**). This enhancement spontaneously regressed six months later, and this lesion was reported as post-treatment changes. CeMRI in this case was false positive

The study included 121 consecutive patients (mean age 37.3 years, age-range 0.5–79.6 years, 61 female). Of these, 70% had undergone previous treatment (12% after complete resection of tumour without known recurrence, 12% after complete resection with known or suspected recurrent disease, 39% after incomplete resection with or without chemo- or radiotherapy, 4% with chemotherapy only, 1% with radiotherapy only, and 2% with chemoradiotherapy) (Fig. 3). The remaining 30% of patients had not started oncological treatment prior to the examination.

Out of the 121 patients, 119 cases (98%) had a lesion on MRI, with 99 being supratentorial and 20 infratentorial. In a subgroup of 118 patients with verified diagnoses, 89 cases (75%) were of tumorous origin (25 LGG; 39 HGG; 25 unknown grade), 19 cases were classified as postoperative changes, 6 as pseudoprogression, and 4 lesions were caused by inflammation. Details of the histopathological findings are shown in the supplement B.

[^18^F]FLT-PET and ceMRI findings were concordant in 119 cases (98%). Contrast enhancement was observed in 71 (59%) patients, while PET activity was noted in 69 patients (57%). Both PET activity and contrast enhancement were present together in 69 patients (57%). Contrast enhancement without PET activity was observed in only 2 patients with postoperative changes. All patients with PET activity also demonstrated concurrent contrast enhancement. The frequency of PET activity and contrast enhancement on MRI across different lesions is summarised in Table 1.

**Table 1:** Frequency of individual lesions on [^18^F]FLT-PET/MRI of the brain and their contrast enhancement and PET activity according to visual assessment

Diseases	*n*	Modality	Positive	Negative	Agree	Disagree
LGG	25	[^18^F]FLT-PET	4	21	25	0
		ceMRI	4	21		
HGG	39	[^18^F]FLT-PET	35	4	39	0
		ceMRI	35	4		
Tumour of unknown grade	25	[^18^F]FLT-PET	13	12	25	0
		ceMRI	13	12		
Inflammation	4	[^18^F]FLT-PET	3	1	4	0
		ceMRI	3	1		
Posttherapeutic changes	19	[^18^F]FLT-PET	8	11	17	2
		ceMRI	10	9		
Pseudoprogression	6	[^18^F]FLT-PET	6	0	6	0
		ceMRI	6	0		

Cohens weighted kappa showed high agreement between ceMRI and [^18^F]FLT-PET (κ = 0.949; *p* < 0.001; 95% CI 0.893–1.006). The Fisher exact test demonstrated an effect of pathology on the agreement between ceMRI and [^18^F]FLT-PET (*p* = 0.0024), though post-hoc tests did not reveal any difference based on adjusted Fisher p-values.

On closer analysis of a subset of 64 patients with verified gliomas, the sensitivity and specificity of both PET and ceMRI findings based on visual evaluation were identical (90% and 84%, respectively) for differentiating LGG from HGG, if contrast enhancement and [^18^F]FLT uptake were considered as hallmarks of HGG. For differentiation of HGG from LGG together with lesions of nontumorous aetiology (inflammatory lesions or post-therapeutic changes) in a subgroup of 93 patients, the sensitivity of both [^18^F]FLT-PET and ceMRI was 90%, while the specificity of [^18^F]FLT-PET was slightly higher (61%) compared to ceMRI (57%). The specificity of [^18^F]FLT-PET was also higher than that of contrast enhancement (41% vs. 34%) in distinguishing HGG from non-tumour lesions (inflammatory lesions or post-therapeutic changes). Table 2.

**Table 2:** Sensitivity and specificity of contrast enhancement and [^18^F]FLT-PET activity of HGG vs. LGG, HGG vs. non-tumour lesions*, and HGG vs. LGG together with non-tumour lesions according to visual assessment

		HGG vs. LGG	HGG vs. LGG together with non-tumour lesions*	HGG vs. non-tumour lesions*
Contrast enhancement	sensitivity	90%	90%	90%
	specificity	84%	57%	34%
[^18^F]FLT-PET	sensitivity	90%	90%	90%
	specificity	84%	61%	41%

*inflammation, pseudoprogression, posttherapeutic changes

Tables 3 and 4 detail SUVmax, T/N ratios, and AUC values with sensitivity and specificity for the calculated thresholds in all verified lesions. Significant differences in SUVmax and T/N ratios were observed between HGG vs. LGG (*p* < 0.001), HGG vs. non-tumour lesions (*p* < 0.01), and HGG vs. LGG together with non-tumour lesion (*p* < 0.001).

**Table 3:** SUVmax and T/N ratio of [^18^F]FLT-PET/MRI for all verified lesions

	SUVmax average ± SD (min – max) [g/ml]	T/*N* ratio average ± SD (min – max)
HGG	1.96 ± 1.40 (0.17–5.57)	11.82 ± 9.31 (1.55–42.89)
LGG	0.56 ± 0.62 (0.11–2.57)	3.20 ± 3.13 (0.73–10.71)
All non-tumour lesion	0.96 ± 0.98 (0.18–4.96)	6.26 ± 6.53 (0.73–26.50)
All non-tumour lesion and LGG	0.76 ± 0.84 (0.11–4.96)	4.81 ± 5.34 (0.73–26.50)

SUV – standard uptake value, SD – standard deviation, HGG – high-grade glioma, LGG – low-grade glioma

**Table 4:** AUC values of SUVmax and T/N ratios including sensitivity and specificity for calculated thresholds in all verified lesions

	SUVmax threshold	SUVmax AUC (sensitivity; specificity)	T/*N* ratio threshold	T/*N* ratio AUC (sensitivity; specificity)
HGG vs. LGG	0.68	0.857 (87.2; 80.0)***	3.33	0.869 (89.7;80.0)***
HGG vs. all non-tumour lesions	1.21	0.762 (66.7; 82.1)**	6.37	0.745 (69.2;75.0)**
HGG vs. all non-tumour lesions and LGG	0.9	0.808 (82.1; 74.1)***	5.28	0.805 (82.1;72.2)***

SUV – standard uptake value, AUC – area under the curve, HGG – high-grade glioma, LGG – low-grade glioma

**p < 0.01

*** *p* < 0.001

## Discussion

Based on the findings of this study, [^18^F]fluorothymidine-PET and contrast-enhanced MRI (ceMRI) demonstrated a remarkably high concordance rate in patients with suspected or known brain tumours. Our study expands upon previous research by demonstrating a high level of agreement between [^18^F]FLT-PET and ceMRI findings, which were simultaneously acquired, thus minimising biases associated with separate imaging sessions.

Overall, [^18^F]FLT-PET showed slightly higher specificity than contrast-enhanced MRI in distinguishing HGG from non-tumour lesions (41% vs. 34%) and in differentiating HGG from a combination of LGG and non-tumour lesions (61% vs. 57%), although these differences were not statistically significant. This finding aligns with observations that [^18^F]FLT requires a disrupted blood-brain barrier for effective penetration into tumour cells. Out of 121 patients, PET activity and contrast enhancement detected on MRI did not match in only 2 cases, where MRI contrast enhancement represented postoperative changes in patients with low-grade gliomas. Moreover, the contrast enhancement in both cases was very subtle, which may explain the undetectable PET activity due to the poorer spatial resolution of PET imaging.

Other studies performed separately on PET and MRI scanner and focused only on high grade gliomas also found a significant correlation between areas of [^18^F]FLT uptake and MRI contrast enhancement on very small number of patients (10 and 23) [18, 19]. In one of those studies, [^18^F]FLT-PET was able to detect 21 of 23 high grade gliomas and was negative only in 2 cases (glioblastoma and anaplastic astrocytoma) with no or moderate contrast enhancement ^19^. Volume of increased [^18^F]FLT-PET further closely correlated with volume of contrast enhancement on MRI [19]. For comparison, [^18^F]FET-PET of the same study group revealed 22 of 23 high grade gliomas, with only 1 anaplastic astrocytoma with contrast enhancement missing. [^18^F]FET-PET was also able to reveal nonenhanced parts of the tumour [19]. Compared to these studies, our work brings several important aspects. In addition to a significantly larger number of subjects, we do not limit ourselves to high grade gliomas, but also to other pathologies representing the spectrum of real-world imaging findings, using state-of-the-art PET/MRI device equipped with advanced technical solutions (LSO crystals, semiconductor amplifiers, Time of Flight (TOF) reconstruction, etc.) and simultaneous data acquisition, which eliminates spatial and temporal bias between PET/CT and follow-up MRI. In another study focused on suspected glioma recurrence, the SUVmax of [^18^F]FLT-PET was significantly higher for recurrence of high-grade gliomas than for postoperative changes (1.77 g/ml vs. 0.40 g/ml), but no difference was observed between initially low-grade gliomas and postoperative changes (SUVmax 0.66 g/ml) [20]. These data were consistent with our results, with SUVmax of HGG 1.96 g/ml and non-tumour lesions (including posttreatment changes, pseudoprogression and inflammation) 0.96 g/ml (*p* < 0.01) with sensitivity and specificity 66.7% and 82.1% for SUVmax and 69.2% and 75.0% for T/N ratio. These results are similar to the reported sensitivity and specificity of anatomical MRI (68% and 77%) and MRI with apparent diffusion coefficient (71% and 87%), but achieve lower sensitivity than other amino acid PET tracers ([^18^F]FET, [^11^C]MET, [^18^F]DOPA) with reported sensitivity of 85–92% and specificity of 67–79% [21].

Our results suggest that quantification of SUVmax may improve the specificity for differentiation of HGG from non-tumour lesions compared to visual evaluation of the radiopharmaceutical uptake (82 and 41% respectively). However, the sensitivity of ROC analysis given by the threshold calculated using Youden’s method was substantially lower compared to visual analysis (67 and 90%), which is suboptimal from the clinical perspective, as a significant number of tumour recurrence lesions might be missed.

ROC analysis also showed that using SUVmax reached the same results in differentiating between HGG and LGG subgroups as the T/N ratio. In some studies, the T/N ratio provided better results than SUVmax in discriminating recurrent gliomas from necrosis [22]. Conversely, other studies have shown that T/N ratio and SUVmax perform similarly in distinguishing metastatic brain lesions and HGG from pseudoprogression ﻿[9, 15]. An advantage of SUVmax is that it eliminates the need to mirror the ROI to the contralateral side of the brain, a task that can be difficult and poorly reproducible, potentially increasing bias.

[^18^F]FLT-PET/MRI is not the method of choice for a wide spectrum of pathologies, which can be confirmed by some meta-analyses [23, 24]. As shown in our study, [^18^F]FLT-PET achieves slightly higher specificity in the detection of high-grade tumours, but in most cases, ceMRI is a comparable method that is cheaper, safer and more accessible. Therefore, [^18^F]FLT-PET should not be considered as a first-line diagnostic modality, and even its role as a complementary test in equivocal MRI findings is questionable.

Given the very high concordance between [^18^F]FLT-PET and ceMRI results, it may be more appropriate to replace [^18^F]FLT with another radiopharmaceutical that does not demonstrate such a strong association with disruption of the blood-brain barrier. Appropriate alternatives could be [^18^F]fluoroethyl-tyrosine (FET) or [^11^C]methionine (MET), both of which are suitable for differentiation of brain tumours or non-tumour lesions, as their uptake is not dependent on blood-brain barrier disruption, making them more suitable for diagnosis of low-grade glioma than [^18^F]FLT [24]. Among the new tracers, FAPI (Fibroblast activation protein inhibitor) has shown promise. In a pilot study on PET/CT, FAPI was able to detect glioblastomas at lower rate than MRI but achieved a 100% positive predictive value [25]. Another option is to use dynamic or pseudo-dynamic [^18^F]FLT-PET [26, 27]. Wardak et al. have demonstrated the significant role of FLT kinetic parameters in predicting overall survival in patients treated for recurrent brain tumours [14, 28]. However, such approaches are technically demanding and are currently not used in a daily practice.

This study has several limitations. As a retrospective study using real-world data without an external control group, its generalisability is limited. The specific interval during which the data for the study were obtained may not fully capture the dynamic nature of lesions, and a minimum one-year follow-up may overlook some cases of slower-growing low-grade gliomas (LGGs).

The decision to use regions of interest (ROI) for measuring SUV, although subject to potential subjectivity, was based on practical reasons and has been used in the methodology of previous studies, [15] ensuring consistency and comparability with existing literature and our internal practices. ROI analysis allowed for specific localisation of PET signals, facilitating the comparison of PET findings with ceMRI, and ensuring reproducibility across imaging sessions and observers.

Histological confirmation was not feasible for all lesions due to ethical and clinical reasons, adding uncertainty to lesion classification. Overlapping definitions of non-tumour lesions, such as postoperative changes and pseudoprogression, could impact specificity. While simultaneous PET/MRI acquisition minimised biases, the spatial resolution of PET imaging is inferior to MRI, limiting the detection of subtle changes.

In summary, our study demonstrated the capability of [^18^F]FLT to distinguish between high-grade and low-grade gliomas and to differentiate tumours from lesions of other origins. However, consistent with previous findings, we have affirmed in our relatively large patient cohort a very high correlation between ceMRI and [^18^F]FLT-PET findings, underscored by simultaneous data acquisition on a hybrid PET/MRI machine. Therefore, [^18^F]FLT-PET findings do not appear to significantly enhance diagnostic accuracy. Conventional MRI, potentially complemented by advanced techniques such as diffusion or perfusion imaging, suffices as a diagnostic method for patients with focal brain lesions compared to [^18^F]FLT-PET.

### Electronic supplementary material

Below is the link to the electronic supplementary material.

Supplementary Material 1

## Data Availability

Data available on request from the corresponding author.
