# Supplementary material for: The relationship between gadolinium enhancement and [18 F]fluorothymidine uptake in brain lesions with the use of hybrid PET/MRI
Source: Cancer Imaging. 2024 Aug 19;24:110. doi: 10.1186/s40644-024-00761-0 (PMC11331680; doi:10.1186/s40644-024-00761-0)
Supplement: Supplementary file 1 — Supplementary Material 1 [file 40644_2024_761_MOESM1_ESM.pdf]

Supplement A - Table 1 – Parameters of MRI sequences

Parameters	Sag T1 SE	Ax T2 FSE	Ax T2* GRE	Ax DWI	Ax 3D ASL	Sag 3D FLAIR ce	Ax T1 SE ce	Ax 3D T1 ce	Sag T1 FLAIR ce
FOV [cm]	24x24	24x24	24x19.2	24x24	24x24	25.6x25.6	24x19.2	24x19.2	24x24
Acq.vox size [mm^2]	0.8x1.1	0.6x0.7	0.8x0.9	1.9x1.9	1.88x1.88	1x1	0.8x1.1	1x1	0.7x0.9
Slice th. [mm]	5	4	4	5	4	1.4	4	1	4
TR/TE [ms]	820/15	6300/102	640/11	6200/77	5200/11	7000/120	800/11	8.2/3.1	3800/24
ETL/Flip ang.	-/-	14/-	-/15°	-/-	-/-	140/-	-/-	-/12	8/-
NEX	1	1	1	1	3	1	1	1	1
ARC/HS	2	2	2	2	-	2x2/1.3	2	2x1	2
Other				b=0; 1000		FatSat<br>TI=1950		BRAVO	TI=960

Sag – Sagittal, Ax – Axial, SE – Spin echo, FSE – Fast Spin Echo, GRE – Gradient Echo, DWI – Diffusion Weighted Imaging, ASL – Arterial Spin Labeling, ce – Contrast Enhancement, FLAIR – Fluid Attenuated Inversion Recovery, FOV – Field Of View, Acq. Vox. Size – Acquisition voxel size, Slice th. – Slice thickness, TR – Repetition Time, TE – Echo Time, ETL – Echo Train Length, Flip ang. – Flip angle, NEX – Number of Exitation, ARC – Autocalibrating Reconstruction for Cartesian imaging (parallel acquisition technique), HS – HyperSense (compressed sensing technique), FatSat – Fat Saturation, TI – Inversion Time, BRAVO – BRAin VOlume.

Supplement B - Histological types of tumors and type of confirmation of the lesion was added to the appendix.

High grade gliomas (n=39; 5 verified by biopsy and 34 by resection – 20 pre and 19 post PET/MRI): 16 glioblastoma, 6 astrocytoma with parts of anaplastic astrocytoma, 6 anaplastic astrocytoma, 4 anaplastic ependymoma, 3 anaplastic oligodendroglioma, 1 pleomorphic xanthoastrocytoma with anaplastic features, 1 oligoastrocytoma with parts of anaplastic astrocytoma, 1 oligodendroglioma with parts of anaplastic oligodendroglioma, 1 diffuse midline glioma H3 K27-altered with progression to high grade glioma

Low grade gliomas (n=25; 3 verified by biopsy and 22 by resection – 14 pre and 11 post PET/MRI): 19 astrocytoma, 4 oligodendroglioma, 1 astrocytoma combined with tumefactive multiple sclerosis, 1 optochiasmatic glioma

Tumors of unknown grade (n=25; histologically verified only in 7 cases – 4 resections, 2 biopsy, 1 CSF cytology): 11 appearance of low grade glioma without histology, 1 adenoid cystic carcinoma, 1 sinonasal angiosarcoma, 1 brain stem vascular malformation, 2 low grade glioma without specification, 1 diffuse leptomeningeal glioneuronal tumor, 2 glioneural tumor not closely specified, 1 juvenile myofibromatosis of the skull, 1 diffuse midline glioma H3 K27-altered, 1 without histological verification due to early death, 1 teratoma of the pineal region, 1 pilocytic astrocytoma, 1 nongerminomatous germ cell tumor of the pineal region

Inflammation (n=4 - 3 verified by follow up and 1 tumefactive multiple sclerosis proven by autopsy) – 1 encephalitis, 2 tumefactive multiple sclerosis, 1 suspected CLIPPERS disease.

All 6 cases of pseudoprogression were verified by follow up. Three cases of postoperative changes were proven by resection, 16 by follow up.

In a 38-year-old patient with a history of resected cystic spongioblastoma at the age of 3 years and now unclear findings in the resection cavity, a definitive diagnosis was not made due to lack of follow up.
